# Supplementary material for: Cost-effectiveness of the TherMax blood warmer during continuous renal replacement therapy
Source: PLoS One. 2022 Feb 3;17(2):e0263054. doi: 10.1371/journal.pone.0263054 (PMC8812918; doi:10.1371/journal.pone.0263054)
Supplement: S1 Table — This supporting table is an more detailed expansion of the model inputs list provided in Table 1 of the main text. This table additionally includes sources for each input along with a description of the source and statistic. (DOCX) [file pone.0263054.s001.docx]

| **Parameter** | **TherMax Warmer** | **Standalone Warmer** | **Reference** |
| --- | --- | --- | --- |
| **Patient Age (yr)** | 65.4 | 65.4 | Internal Data on File |
| **Life years remaining**  **(US population age 65)** | 17.5 | 17.5 | USRDS 2020 – Average life-years remaining among dialysis patients and general US population (18)  KFF 2020 – Sex distribution in the US by Age group (19) |
| **Life years remaining**  **(ESRD on dialysis age 65)** | 7.8 | 7.8 | USRDS 2020 – Average life-years remaining among dialysis patients and general US population (18) |
| **Chronic Dialysis (%)** | 21.8 | 21.8 | Wald 2013 – Proportion of patients on long-term dialysis at 90s following CRRT for AKI (17) |
| **Hypothermia** | 0.345 | 0.719 | Internal Data on File |
| **Mortality** |  |  |  |
| Among hormothermic | 0.400 | 0.400 | Ethgen 2015 - ICU mortality among AKI patients on CRRT (assumed normothermia) (15) |
| Among hypothermic | 0.592 | 0.592 | Erkens 2019 - SAPS-2 adjusted intra-ICU mortality (RR = 1.48) among patients with hypothermia (<36C) at time of admission (16) |
| **ICU Length of Stay (days)** |  |  |  |
| Among cormothermic | 12.0 | 12.0 | Uchino 2007 - ICU length of stay among patients with AKI treated with CRRT, international cohort (20) |
| Among hypothermic | 13.7 | 13.7 | Inaba 2009 - Mean difference in LOS between hypothermia and normothermia at admission to post-op ICU = 1.72 days (12) |
| **Duration of CRRT (days)** | 7.0 | 7.0 | Ethgen 2015 - days on CRRT (15) |
| **Costs** |  |  |  |
| Cost per inpatient day | $5,611 | $5,611 | Kramer 2015 - Mean total cost ($16,353) / mean ICU LOS (3.3 days) in the US from 2012-2016, inflated to from 2013 to 2020 (21) |
| Cost of RRT per day | $961 | $961 | Ethgen 2015 - acute CRRT cost per day, adjusted to CPI 2020 (15) |
| Device Cost | $50,000 | $38,000 | Baxter 2020 – US List price, PrisMax and PrismaFlex, respectively (22) |
