# Supplementary material for: Cost-effectiveness of the TherMax blood warmer during continuous renal replacement therapy
Source: PLoS One. 2022 Feb 3;17(2):e0263054. doi: 10.1371/journal.pone.0263054 (PMC8812918; doi:10.1371/journal.pone.0263054)
Supplement: Attachment — Submitted filename: Response to Reviewers.docx [file pone.0263054.s002.docx]

**Response to Reviewers**

Reviewer #1: The authors described a novel device to reduce risk of hypothermia during CRRT, the topic is interesting in the ICU setting, perhaps the manuscript needs major revision and the conclusions are not completely supported by data.

**Major revision:**
- Please move the definition of hypothermia in the methods.

The definition of hypothermia is already in the methods under the subsection “Health outcomes and costs”. Since Hypothermia is a heath outcome of CRRT, we believe this is an appropriate place for the definition. We hope the reviewer agrees.

- Please describe in the methods how data transfer has been made.

Communications between authors is not an inherent nor necessarily reproducible part of the methods. It would be unorthodox to publish such communications, but for the reviewer’s own knowledge, the data was transferred in a zipped folder via encrypted e-mail, then transferred to a centralized remote sever for analysis. We hope this will suffice.

- Is there any chance to record the temperature every minute? Did the authors consider in the analysis a single value recorded or at least an hour of hypothermia?

Unfortunately, recordings each minute would have been unfeasible since the temperatures were recorded by nursing staff who were not available for each patient every minute. In most cases, there was only a single temperature value recorded each hour and in cases where there was more than one record per hour, it was by error.

- Page 4 lines 90-91: The authors mentioned as inclusion criteria CRRT for at least 1 hour, I think the duration of the treatment is too short to affect the outcome, please discuss about that.

The intention was to capture all patients who were treated with CRRT since the exposure risk is CRRT. Patients treated for short durations may be at lower risk for hypothermia, but the risk is not 0, and should therefore be included in the analysis. Furthermore, classification and inclusion of said low-risk/short-duration patients into a binary (yes/no) CRRT exposure may introduce misclassification, but misclassification is non-differential with respect to warmer type and unlikely to introduce bias into the results except possibly toward null association. We have added this limitation to the discussion.

-Please provide the mean duration of the CRRT in the 2 groups.

Thank you for the suggestion – We have added median hours on CRRT per patient-stay (the distribution was heavily right-skewed, so mean was not appropriate).

- Page 5, line 98: Recording temperature from different sites could be a limit of the study, please discuss about that.

All methods used to measure temperature are understood to operate in a range of 0.1 Celcius accuracy. We have added more detail in the methods about this, but given the accuraacy and precision of these methods, there is no expected impact on the results.

- Table 1: Please write the exact p value instead of “not significant” or “p<0.05”.

Thank you, the p-values have been added to Table 1.

- Table 1: Number of treatments in the 2 groups are not in accordance with the text, in the table are 310 vs 32, in the manuscript the authors mentioned 526 patients treated with CRRT, it’s difficult for me to understand.

Thank you, the sample sizes reported in the table were left behind in error from a previous analysis. The sample sizes in the table have been updated to reflect the current analysis.

- Table 1: The comparison between 310 and 32 treatments could be a statistical issue.

There is no known statistical error or bias introduced by comparing two groups of unequal size given the statistical methods described. Two-sample tests for comparisons of means (t-test) and proportions (chi-square test) are robust to this.

- In the introduction the authors described the potential complications of the hypothermia, such as arrhythmia, coagulopathy, but they did not mention kind and rate of complications in the study.

While these events are clinically relevant in understanding the consequences of hypothermia, the specific event rates of those complications are not relevant to the scope of this study.

- Page 11: It’s difficult for me to understand this limit “the current data was obtained over 10 months”, the authors wrote in the methods that they included patients between 2006 and 2020, please clarify.

The limitations section states “the current **Prismax/Thermax** data was obtained over 10 months”, which is true. The sentence just before this statement speaks to the comparator group, being previous generation standalone warmer: “the historic group was larger and spanned over 12 years”. We have highlighted this in the methods section with some additional language to clarify.

- In the conclusion: the statement “TherMax reduced the risk of mortality associated with hypothermia” is not supported by data, in table 2 I can see the same mortality for hypothermia in both groups 59.2% and I think it’s quite hard to assume that the mortality is due to hypothermia, please make a comment.
The risk of mortality among patients with hypothermia is not different between Thermax and previous generation warmers. However, the risk of hypothermia is different. Thus, the simulated differential risk of hypothermia is driving the simulated difference in mortality. We have modified the Table 2 parameter names (column 1) and the language in the conclusions to clarify this.

With that said, the Markov simulation models only the difference in risk of mortality among patients with and without hypothermia. Thus, the model ensures that the death can only be due to hypothermia, assuming no confounding of the model’s inputs. Since the input for that difference in risk was adjusted for known confounders, it is unlikely to be a result of some other confounding factor and therefore is most likely due to hypothermia or complications of hypothermia.

With respect to establishing certain causality, it is important to highlight that the conclusions state “TherMax… is ***associated with less risk*** of hypothermia”, which is consistent with our results. Association is not causation, which is why we were careful not to use words or phrases like “reduced”, “caused”, or “resulted in” when describing the main findings of our study.

Please let us know if this comment will suffice.

**Minor revision:**
- Please type “TherMax” in the same way in the manuscript (“Thermax” vs “TherMax”).

Thank you, this has been addressed.

**Other revisions identified by authors:**

Table 1- the patient group column headings were backward – This has been fixed

Line numbers had stopped halfway through in error due to section break – This has been fixed
